# Lupus erythematosus-specific bullous lesions: A case report

**DOI:** 10.1097/MD.0000000000047808

**Published:** 2026-03-27

**Authors:** Shuiling Li, Minghai Zhang, Yuanmei Nie, Xiaoyan Yang

**Affiliations:** a The Fourth Affiliated Hospital of Anhui Medical University, Hefei, Anhui Province, China.

**Keywords:** diagnosis, hydroxychloroquine, lupus erythematosus-specific bullous lesions, methylprednisolone

## Abstract

**Rationale:**

Lupus erythematosus-specific bullous lesions (LE-SBL) is a rare cutaneous phenotype of lupus erythematosus, characterized by tense vesicles or bullae that readily mimic other primary bullous disorders.

**Patient concerns:**

A 79-year-old man presented with an abrupt onset of extensive tense bullae and erosions on the trunk, extremities, and oral mucosa, accompanied by pruritus and pain; no systemic involvement was detected.

**Diagnoses:**

Histopathology revealed a subepidermal blister with a predominantly lymphocytic infiltrate. Enzyme-linked immunosorbent assay for anti-type VII collagen antibodies was negative. Serologic studies demonstrated a markedly elevated anti-Ro-52 antibody titer (>500 U/mL) and a weakly positive antinuclear antibody (1:80, nucleolar pattern). A diagnosis of LE-SBL was established.

**Interventions:**

Intravenous methylprednisolone combined with oral hydroxychloroquine was administered, supplemented by symptomatic care.

**Outcomes:**

After systematic treatment, the patient’s blisters subsided and the eroded areas healed, with no new skin lesions observed during the follow-up period.

**Lessons:**

This study provides a comprehensive analysis of LE-SBL and further refines current understanding of the disease. Integration of histopathology, direct/indirect immunofluorescence, and serologic testing is essential to distinguish this entity from other bullous dermatoses. Early institution of glucocorticoids plus hydroxychloroquine achieves rapid disease control. This report offers an important reference for clinicians encountering similar cases.

## 1. Introduction

Currently, the diagnosis of lupus erythematosus-specific bullous lesions (LE-SBL) primarily relies on clinical manifestations, histopathological examination, direct immunofluorescence (DIF), indirect immunofluorescence, and enzyme-linked immunosorbent assay. This disease requires differential diagnosis from conditions such as pemphigus vulgaris (PV), bullous pemphigoid (BP), dermatitis herpetiformis (DH), and epidermolysis bullosa acquisita (EBA). A report by Kristin N. Smith et al^[1]^ confirmed that LE-SBL can occur independently or present as a cutaneous manifestation of systemic lupus erythematosus (SLE). Treatment primarily centers on antimalarials, glucocorticoids, and various immunosuppressants.^[2]^ Among these treatments, dapsone is considered a first-line option for managing bullous skin lesions; however, in cases where patients experience adverse effects or inadequate response, substitution with hydroxychloroquine or other immunosuppressants may be warranted.^[2]^ To date, there is a lack of comprehensive reporting on LE-SBL both domestically and internationally. The scarcity of literature has become a major bottleneck limiting in-depth clinical understanding and standardized diagnosis and treatment. A limited number of case reports on LE-SBL have been identified only through searches of Chinese National Knowledge Infrastructure, Wanfang, and PubMed,^[1]^ suggesting that this condition remains an under‑recognized entity, requiring more evidence‑based data to refine diagnostic and therapeutic strategies. This article presents a case of LE‑SBL without systemic involvement. The patient’s skin lesions improved rapidly after treatment with methylprednisolone combined with hydroxychloroquine. By systematically reviewing the clinical presentation, diagnostic workup, and treatment response, this case provides a reference for the diagnosis and management of LE‑SBL.

## 2. Case summary

*Patient information*: Gender: male. Age: 79 years. Chief complaint: generalized cutaneous eruption associated with pruritus for 20 days. History of present illness: 20 days prior to admission, the patient developed painful erosions on the lips without identifiable precipitating factors. He sought care at a local hospital and received intravenous therapy (details unknown), with no appreciable improvement. Over the ensuing days, erythematous macules and papules appeared on the trunk and extremities, evolving into vesicles and tense bullae accompanied by mild pruritus. After the patient manually ruptured several bullae, the lesions oozed serous fluid, and became exquisitely tender. Topical ointments (unspecified) produced partial drying and crusting; nevertheless, new lesions continued to emerge. Ten days before the current admission, the patient presented to our hospital’s Department of Stomatology, where treatment with sodium bicarbonate mouth rinses and topical erythromycin ointment was initiated. Oral, labial, and lingual erosions gradually improved; however, the generalized bullous eruption persisted. The patient was subsequently referred to our department for further evaluation and was admitted under a provisional diagnosis of pemphigus. During the entire course, the patient denied fever, headache, dizziness, chest tightness, dyspnea, cough, or sputum production. Appetite and sleep remained adequate; urinary output was normal. Bowel movements were difficult, but no significant weight loss was reported.

*Past medical history*: The patient had previously enjoyed good health. There was no documented history of hypertension, diabetes mellitus, or hypersensitivity reactions to food or drugs.

*Physical examination*: Vital signs: temperature 36.3 °C, pulse 74 beats · min^−1^, respiration 18 breaths · min^−1^, blood pressure 127/64 mm Hg, body weight 63 kg.

**Figure 1.**
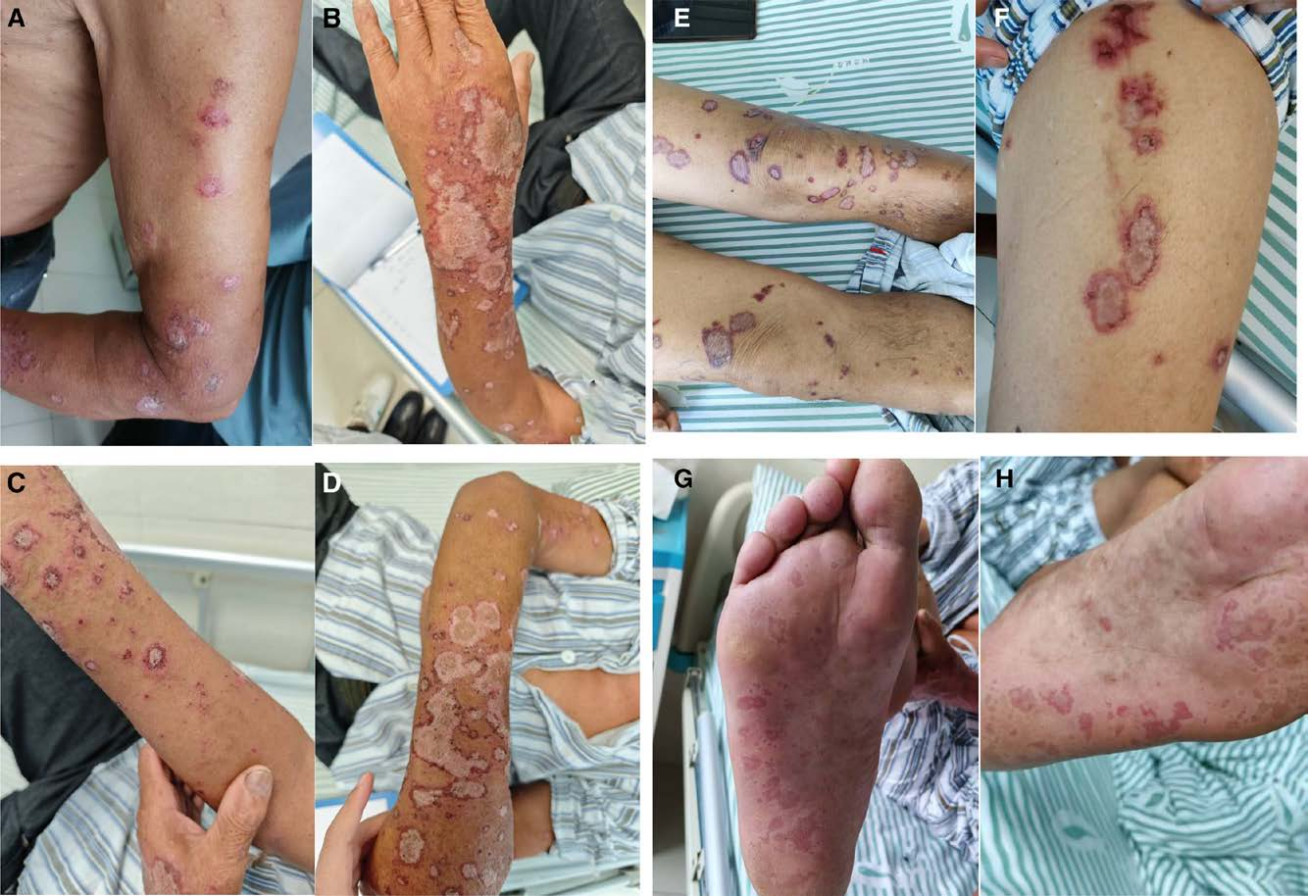
(A–F) Skin lesions at the time of patient admission. (G and H) Newly developed lesions on the plantar surfaces of both feet observed on August 4, 2025.

*Dermatologic findings*: Perioral region: scattered erosions without appreciable exudate. Trunk and extremities: diffusely distributed, faintly erythematous macules, patches, and erosions, many arranged in annular or polycyclic configurations; Nikolsky sign negative. Portions of the lesions exhibited dry hemorrhagic crusts. On both upper extremities, numerous pinpoint- to millet-sized erythematous and skin-colored papulovesicles and tense vesicles were densely clustered (Fig. 1A–H).

*Laboratory and ancillary investigations*: Complete blood count, urinalysis, stool routine, liver and renal function panels, serum electrolytes, lipid profile, anti-hepatitis C antibody, syphilis and human immunodeficiency virus screening, male tumor-marker panel (α-fetoprotein and abnormal prothrombin), quantitative immunoglobulins, rheumatoid factor and anti-cyclic citrullinated peptide antibodies, complement cascade (C_3_, C_4_, and CH_50_), total IgE, vasculitis screen (7 analytes), anticardiolipin antibodies (IgA, IgM, and IgG), and anti-type VII collagen antibodies (IgA, IgG, and IgM) were all within normal limits or negative.

*Inflammatory markers*: High-sensitivity C-reactive protein 6.15 mg/L; erythrocyte sedimentation rate 18 mm/h (reference ≤ 15 mm/h). Coagulation profile: D-dimer 1.15 µg/mL (reference ≤ 0.55 µg/mL). Extractable nuclear antigen panel: anti-Ro-52 antibody >500.00 U/mL (reference ≤ 20 U/mL); all other profiled antibodies negative. Antinuclear antibody (ANA) quantitative assay: 112.81 U/mL (reference ≤ 20 U/mL). Anti-double-stranded DNA antibodies: negative. Anti-Smith antibodies: negative. ANA titer: 1:80, weakly positive; predominant pattern: nucleolar. Hepatitis B serological profile: positive for hepatitis B surface antigen, positive for hepatitis B e antibody, positive for hepatitis B core antibody (HBcAb). Quantitative hepatitis B 5-parameter assay: hepatitis B surface antigen quantitative level: 34.700 IU/mL (reference range: 0–0.05 IU/mL), hepatitis B e antibody level: 0.003 COI (reference range: >1 COI), hepatitis B core antibody level: 0.008 COI (reference range: >1 COI), high-sensitivity hepatitis B virus DNA quantitative PCR: 4.27E+04 IU/mL (reference range: <20 IU/mL) bacterial culture (oropharynx): normal commensal flora isolated.

**Figure 2.**
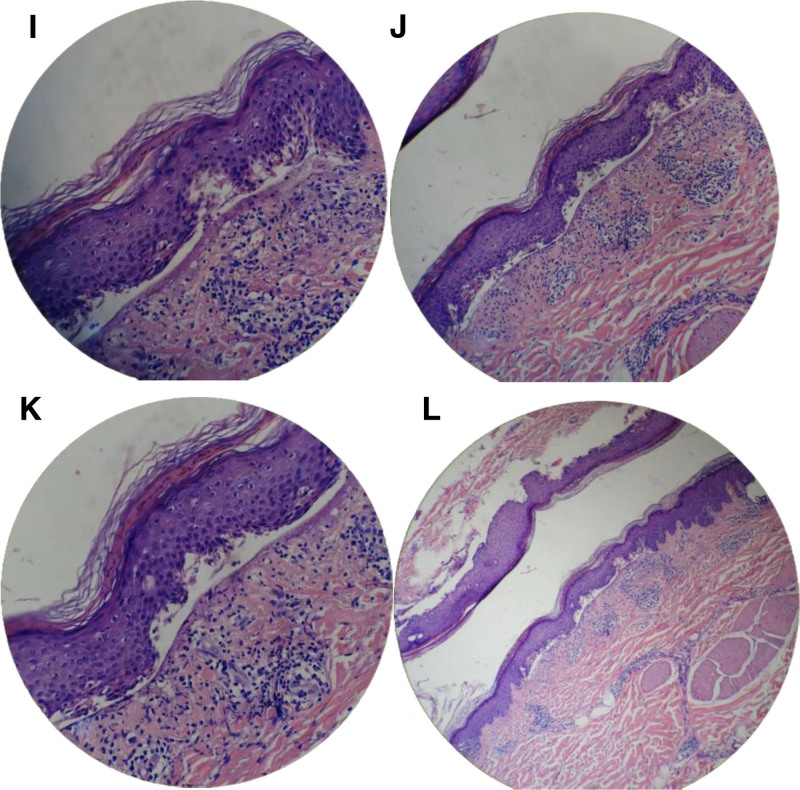
(I–L). Histopathological examination of the skin lesions reveals: clefting (bullae formation) is observed between the epidermal basal layer and the dermal papillary layer. The bullae contain infiltrating neutrophils, and the base of the vesicles shows infiltration of acute and chronic inflammatory cells (H&E staining, ×100).

**Figure 3.**
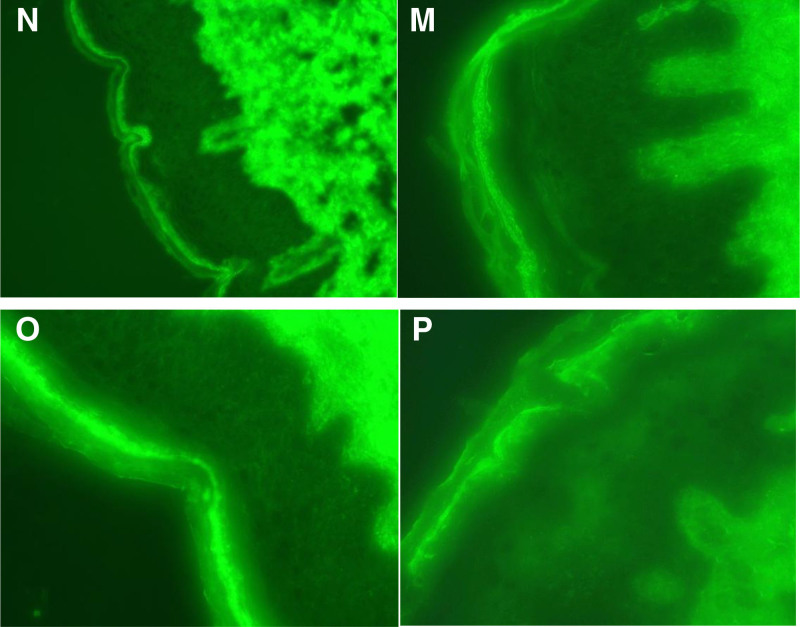
(M–P) Direct immunofluorescence (DIF) of the skin lesions shows: no significant immune complex deposition is observed.

Electrocardiography showed no significant abnormalities. Abdominal and urogenital ultrasonography revealed hepatic steatosis, bilateral renal cysts, bilateral nephrolithiasis, and benign prostatic hyperplasia with calcification. Non-contrast chest computed tomography demonstrated interstitial inflammation in the right middle lobe and both lower lobes, multiple small pulmonary nodules, emphysematous changes, and mildly enlarged mediastinal and bilateral axillary lymph nodes. Histopathology of a freshly developed bulla on the anterolateral right thigh exhibited basal-cell hydropic degeneration and a subepidermal cleft corresponding to bulla formation. The blister cavity contained a sparse lymphocytic infiltrate, and the underlying dermis displayed a mixed acute and chronic inflammatory infiltrate (Fig. 2). DIF of the same biopsy revealed no discernible deposition of immunoglobulins or complement components along the dermo-epidermal junction (Fig. 3).

*Principal diagnosis*: LE-SBL. Principal therapeutic interventions (detailed in Table 1).

**Table 1:** The patient’s clinical progression and detailed management process.

Time	Clinical manifestations	Key interventions	Outcomes
July 09, 2025	Oral mucosal ulceration with associated pain and discomfort.	The patient then sought care at a local hospital, where intravenous therapy was administered; the specific agents used are unavailable.	Despite the intravenous therapy administered at the local hospital, the patient’s symptoms failed to improve significantly. Subsequently, discrete erythematous macules and papules developed over the extremities and trunk; these lesions rapidly evolved to form scattered vesicles and bullae with mild associated pruritus.
July 19, 2025	Oral, labial, and lingual ulcerations were observed.	The patient was referred to the Department of Oral Medicine at our hospital, where treatment was initiated with sodium bicarbonate mouth rinses and topical erythromycin ointment.	Ulcerations of the oral cavity, lips, and tongue gradually resolved; however, the generalized skin eruption with vesicles and bullae remained unimproved.
Admitted on July 29, 2025	A generalized pruritic eruption has been present for 20 days.	A skin biopsy was performed under local anesthesia, targeting a newly formed vesicle on the anterolateral aspect of the right thigh. Empiric therapy was then instituted with intravenous methylprednisolone 40 mg once daily, supplemented by intravenous calcium gluconate 10 mL once daily plus vitamin C 2 g once daily. Adjunctive measures included oral levocetirizine hydrochloride 5 mg nightly, sustained-release potassium chloride 0.5 g 3 times daily, and rabeprazole sodium enteric-coated tablets 10 mg twice daily.	The patient’s condition remains under active surveillance.
July 30, 2025	Hepatitis B serological screening revealed the “e-minus” pattern (HBsAg-positive, anti-HBe-positive, anti-HBc-positive), consistent with inactive chronic hepatitis B infection.	The consulting infectious-disease specialist advised the following investigations: quantitative hepatitis B 5-marker panel, ultrasensitive hepatitis B virus DNA quantification, serum α-fetoprotein, and des-γ-carboxy prothrombin levels.	Pending the results of the above investigations, further diagnostic and therapeutic decisions will be formulated accordingly.
July 31, 2025	New lesions have appeared on the extremities. Histopathological and direct immunofluorescence examination of the surgical specimen (Figs. 2 and 3), together with the returned serological studies, establish the diagnosis as a LE-SBL.	(1) For the newly erupted lesions: add oral hydroxychloroquine sulfate 0.2 g once daily for its anti-inflammatory effect, and apply topical hydrocortisone butyrate cream twice daily for anti-inflammatory and antipruritic therapy. (2) Upon completion of the hepatitis B work-up, the infectious-disease consultant recommended initiating oral entecavir 0.5 mg once daily for antiviral treatment.	The patient’s condition remains under active surveillance.
August 03, 2025	New lesions have appeared on both plantar surfaces, accompanied by swelling and pain on walking (Fig. 1G and H).	The intravenous methylprednisolone regimen was therefore escalated to 40 mg once daily plus an additional 20 mg every evening.	The patient’s condition remains under active surveillance.
August 07, 2025	The patient reported that the generalized eruption had become darker and drier, without new lesions, and that the plantar lesions were no longer tender or swollen. Physical examination revealed: the angular cheilitis had dried and re-epithelialized. Diffuse, faintly erythematous macules and patches with overlying crusts were present on the extremities and trunk; most displayed an annular configuration with central atrophy and elevated borders. The biopsy incision on the right thigh was well approximated. Dark-red macules were noted on both plantar surfaces.	The disease is now under control; the current therapeutic regimen is to be continued unchanged.	The patient’s condition remains under active surveillance.
August 11, 2025	The patient reported that the generalized eruption had become darker and flatter, with no pruritus, pain, or new lesions. On examination: diffuse, dark-red to brown macules and patches with post-inflammatory hyperpigmentation were evident over the extremities and trunk. The biopsy incision on the right thigh remained well approximated. Dark-red macules persisted on both plantar surfaces.	After 4 consecutive days of stable disease, the methylprednisolone regimen was modified to intravenous 40 mg once daily plus oral 4 mg every evening.	Following the corticosteroid taper, no recurrence of the generalized eruption has been observed.
August 14, 2025	No new lesions have appeared following corticosteroid tapering. The patient remains free of pruritus or pain, and the generalized eruption has continued to darken and flatten. Physical examination showed diffuse, dark-red to brown macules and patches with post-inflammatory hyperpigmentation distributed over the extremities and trunk; the biopsy incision on the right thigh was well approximated, and dark-red macules were noted on both plantar surfaces (Fig. 4).	The patient was discharged with the following maintenance regimen: main therapy Methylprednisolone tablets 28 mg orally after breakfast, 12 mg after lunch, and 4 mg after dinner. Adjunctive therapy rabeprazole sodium enteric-coated tablets 10 mg orally twice daily before meals. Potassium chloride sustained-release tablets 0.5 g orally 3 times daily. Levocetirizine hydrochloride tablets 5 mg orally once nightly. Calcium carbonate–vitamin D_3_ tablets (600 mg elemental calcium/125 IU vitamin D_3_ per tablet) 1 tablet orally once daily. Entecavir 0.5 mg orally once daily.	The patient remains under regular follow-up.
August 23, 2025	No new lesions have developed. Physical examination reveals numerous dark-red, pink, and gray-white macules and patches distributed over the extremities and trunk. Repeat complete blood count and comprehensive metabolic panel show no significant abnormalities.	Medication adjustment: methylprednisolone tablets 12 mg 3 times daily (tid). All other treatments remain unchanged.	The patient remains under regular follow-up.

LE-SBL = lupus erythematosus-specific bullous lesions.

**Figure 4.**
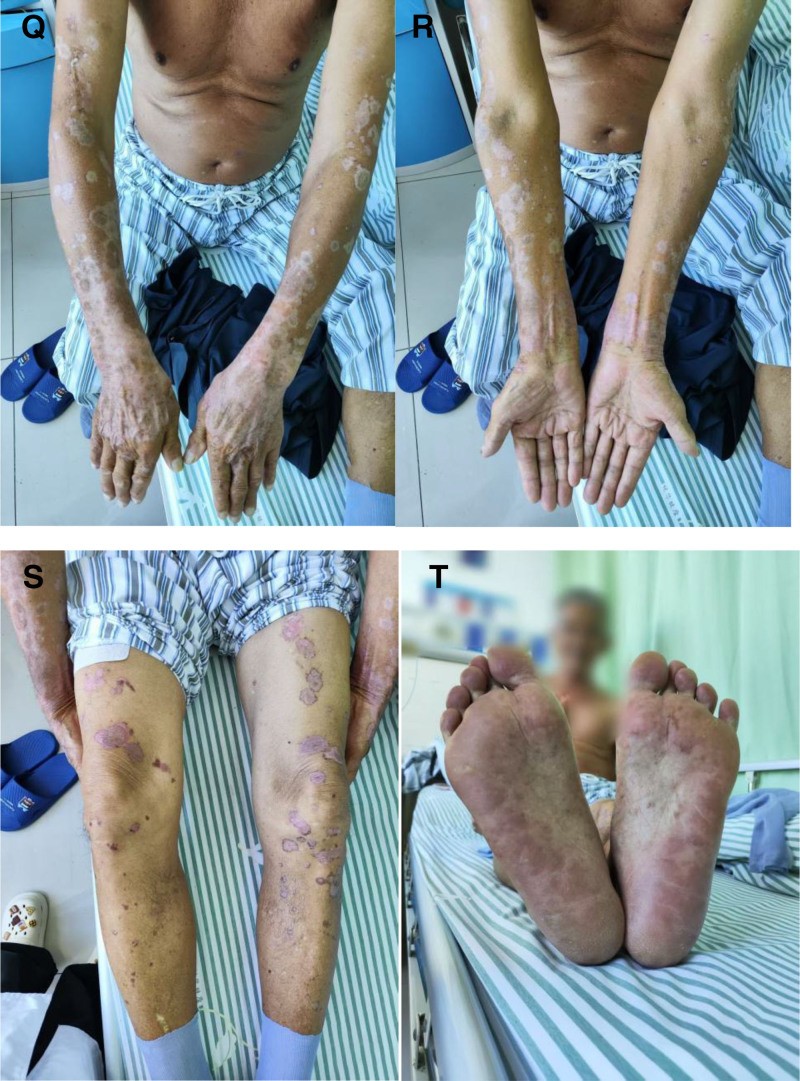
(Q–T) Skin lesions at the time of patient discharge on August 14, 2025.

Upon admission, the patient underwent comprehensive laboratory investigations and, under local anesthesia, a skin biopsy for routine histopathology and DIF. Empirical therapy was immediately initiated with intravenous methylprednisolone 40 mg once daily. Adjunctive measures comprised intravenous calcium gluconate 10 mL once daily plus vitamin C 2 g once daily, oral levocetirizine hydrochloride 5 mg at night, sustained-release potassium chloride 0.5 g 3 times daily, and enteric-coated rabeprazole sodium 10 mg twice daily for gastro-protection. After review of the evolving clinical picture and confirmatory investigations, oral hydroxychloroquine sulfate 0.2 g once daily was added for its anti-inflammatory and immunomodulatory properties, together with topical hydrocortisone butyrate cream for anti-inflammatory and antipruritic effect. Methylprednisolone dosage and dosing schedule were then titrated dynamically in accordance with disease activity. The skin lesions healed well after treatment (Fig. 4Q–T).

## 3. Discussion

LE-SBL are a localized inflammatory skin disease primarily driven by focal inflammation and cytokine cascade reactions.^[1]^ LE-SBL typically presents as tense blisters arising on lupus erythematosus lesions, accompanied by marked erythema and inflammatory infiltration. Approximately 75% of SLE patients may develop cutaneous lesions; however, only about 1% progress to bullous changes, with a predominance among women of childbearing age.^[3]^ Bullous lesions in lupus erythematosus involve 2 distinct pathogenic mechanisms: the first is LE‑SBL resulting from interface dermatitis, and the second is bullous systemic lupus erythematosus (BSLE), which is mediated by autoantibodies and characterized by disruption of the dermo‑epidermal junction.^[1]^ BSLE is closely associated with antitype VII collagen antibodies and is frequently accompanied by multisystem involvement. Patients with BSLE meet the classification criteria for SLE and often present with multisystem damage such as lupus nephritis and arthritis. In contrast, LE‑SBL is typically not associated with systemic involvement, with lesions primarily confined to the skin. Therefore, the presence or absence of systemic manifestations serves as a key criterion for their clinical differentiation. In LE‑SBL, serological tests show significantly lower positivity rates for ANA and anti‑dsDNA antibodies. Indirect immunofluorescence testing for anti‑type VII collagen antibodies is negative. Histologically, the findings are predominantly lymphocytic infiltration, with clefts located above the lamina densa, accompanied by epidermal necrosis, interface reaction, and lymphocytic infiltration around blood vessels and appendages. DIF demonstrates granular deposition of IgG at the basement membrane zone.^[1]^ Among these, the most discriminative indicator is the anti-type VII collagen antibody.^[4–7]^

LE-SBL also requires differentiation from the following conditions: first, PV: PV exhibits acantholysis and anti-Dsg3 antibodies. The present case showed no acantholysis and lacked serological evidence of anti-Dsg3. Second, BP: BP presents with subepidermal blisters, and salt-split skin immunofluorescence demonstrates IgG deposition on the epidermal side. In this case, the cleft was located above the lamina densa. Third, DH: DH is characterized by IgA deposits in the dermal papillae and typically involves extensor surfaces. No dermal papillary IgA deposits were observed in the pathology of this case. Fourth, EBA: EBA is positive for anti-type VII collagen antibodies and often results in scarring, neither of which was present in this case.

LE‑SBL is clinically uncommon, and currently, there is no standardized therapeutic protocol. The management currently draws upon treatment approaches for bullous‑related conditions such as BSLE. The “2021 Guidelines for the Diagnosis, Treatment, and Long‑Term Management of Cutaneous Lupus Erythematosus” recommend dapsone in combination with prednisone as a first‑line treatment regimen, which typically demonstrates significant efficacy.^[2,8]^ In patients with glucose-6-phosphate dehydrogenase deficiency or those carrying the HLA‑B*13:01 allele, the use of dapsone may induce acute hemolytic reactions.^[9]^ Therefore, it is recommended that patients undergo glucose-6-phosphate dehydrogenase activity testing and HLA‑B*13:01 gene screening prior to initiating dapsone therapy.^[2]^ For patients who exhibit an inadequate response or intolerance to dapsone therapy, glucocorticoids or combination therapy with antimalarial agents (such as hydroxychloroquine) may be considered. Antimalarial agents also help control underlying lupus activity. The EUSCLE study,^[10]^ which included 413 patients with CLE, demonstrated that systemic glucocorticoids showed superior clinical efficacy compared to other systemic therapeutic agents. Furthermore, studies by Yokogawa et al^[11]^ and Bezerra et al^[12]^ have confirmed that antimalarial drugs achieve favorable therapeutic outcomes in CLE patients with severe skin lesions. Meanwhile, the guidelines^[2]^ recommend systemic glucocorticoids combined with antimalarial agents as first‑line therapy for CLE patients with severe skin lesions. Given that dapsone was unavailable at our institution, the patient in this case was treated with a regimen of methylprednisolone combined with hydroxychloroquine, which yielded significant therapeutic efficacy. The patient remains under follow‑up; no new rashes have emerged during steroid tapering, and the condition has remained stable.

Based on the symptoms, signs, and relevant laboratory findings, the patient (an elderly male) was diagnosed with LE-SBL. The diagnostic challenge lies in differentiating this condition from BSLE, as both share highly similar clinical presentations. Distinguishing between them requires comprehensive evaluation, followed by the formulation of an individualized treatment strategy upon definitive diagnosis. BSLE was ruled out because the patient did not meet the diagnostic criteria for SLE and tested negative for anti‑type VII collagen antibodies. Following a systematic review of the literature and in consideration of the patient’s clinical condition, we adjusted the dosage of methylprednisolone. Following treatment, the patient’s skin lesions resolved. This case demonstrates successful disease control with a “glucocorticoid + hydroxychloroquine” regimen, challenging the conventional guideline‑recommended first‑line approach of “glucocorticoid + dapsone.” The efficacy and safety of the “glucocorticoid + dapsone” regimen in treating LE-SBL require further validation through multicenter studies.

## 4. Description

To protect patient privacy, all identifiable information was de-identified, and facial features in photographs were masked. Written informed consent was obtained from the patient (File S1, Supplemental Digital Content, https://links.lww.com/MD/R437), authorizing the use of his/her clinical data under these privacy safeguards for the preparation and publication of this manuscript; the patient also reviewed and approved the final version of the manuscript. This retrospective case analysis utilized fully anonymized medical records; according to the Ethics Committee policy of the Fourth Affiliated Hospital of Anhui Medical University, additional ethical approval was waived for anonymized retrospective studies.

## Author contributions

**Conceptualization:** Shuiling Li, Minghai Zhang, Yuanmei Nie, Xiaoyan Yang.

**Data curation:** Shuiling Li, Minghai Zhang, Yuanmei Nie, Xiaoyan Yang.

**Formal analysis:** Shuiling Li, Minghai Zhang.

**Funding acquisition:** Shuiling Li.

**Investigation:** Shuiling Li.

**Methodology:** Shuiling Li.

**Project administration:** Shuiling Li.

**Resources:** Shuiling Li.

**Software:** Shuiling Li.

**Supervision:** Shuiling Li.

**Validation:** Shuiling Li.

**Visualization:** Shuiling Li.

**Writing – original draft:** Shuiling Li.

**Writing – review & editing:** Shuiling Li.

## Supplementary Material


